# Redo mitral valve replacement with annular reconstruction of left atrial dissection following mitral valve replacement for infective endocarditis: a case report

**DOI:** 10.1186/s44215-025-00188-4

**Published:** 2025-01-15

**Authors:** Hiroki Tada, Junya Yokoyama, Akinobu Otani, Keiwa Kin, Yukitoshi Shirakawa

**Affiliations:** Department of Cardiovascular Surgery, Osaka General Medical Center, Osaka, 558-8558 Japan

**Keywords:** Left atrial dissection, Infective endocarditis, Redo MVR with annular reconstruction

## Abstract

**Background:**

Left atrial dissection is a rare and occasionally fatal complication of cardiac surgery and is defined as the creation of a false chamber through a tear in the mitral valve annulus extending into the left atrial wall. Some patients are asymptomatic, while others present with various symptoms, such as chest pain, dyspnea, and even cardiac arrest. Although there is no established management for left atrial dissection, surgery should be considered in patients with hemodynamic disruption. Herein, we report a case of left atrial dissection managed using redo mitral valve replacement (MVR) with annular reconstruction.

**Case presentation:**

A 60-year-old man presented to our hospital with bilateral lower-extremity purpura and cognitive decline. Blood tests showed an elevated inflammatory response, and blood culture revealed *Streptococcus mitis*. Transesophageal echocardiography (TEE) revealed severe mitral regurgitation with vegetation on both the anterior and posterior leaflets, and infective endocarditis was diagnosed. We performed minimally invasive cardiac surgery-MVR through a right mini thoracotomy using Epic mitral valve 29 mm (Abbott Laboratories, Green Oaks, IL, USA). On postoperative day (POD) 2, the patient was discharged from the intensive care unit (ICU). On POD 3, sudden cardiac arrest occurred; we started cardiopulmonary resuscitation and urgently inserted a peripheral venoarterial extracorporeal membrane oxygenation (VA-ECMO) cannula. Contrast-enhanced computed tomography revealed extravasation from the posterior wall of the left atrium. Therefore, we performed an emergency median sternotomy, controlled the bleeding from the posterior wall of the left atrium, and returned the patient to the ICU with gauze packing under VA-ECMO. Two days later, when the gauze was removed, TEE revealed a false lumen on the left atrial wall, and left atrial dissection was diagnosed. Accordingly, we performed annular reconstruction with bovine pericardium to close the entry point and, in succession, redo MVR with a bioprosthetic Epic mitral valve 27 mm. The postoperative course was uneventful. The patient was transferred to a rehabilitation hospital on POD 74.

**Conclusion:**

We report a case of left atrial dissection following MVR. The complex lesion was successfully repaired using redo MVR with annular reconstruction.

## Background

Left atrial dissection, a rare complication of cardiac surgery, is defined as the creation of a false chamber through a tear in the mitral valve annulus that extends into the left atrial wall. Left atrial dissection occurs in 0.16% of patients after mitral valve surgery [1]; the risk factor is tissue fragility, such as that seen in infective endocarditis (IE), mitral annular calcification, and corticosteroid users [2].

The pathogenesis of this condition can lead to hemodynamic disturbances. Patients with hemodynamic instability reportedly require surgical treatment, such as evacuation of the hematoma, identification of the entry point, and obliteration of the false lumen [1], but there is no clear consensus on its management. To the best of our knowledge, there have been no reports of redo mitral valve replacement (MVR) with annular reconstruction for left atrial dissection following MVR.

Herein, we present a case of left atrial dissection managed with redo MVR and annular reconstruction.

## Case presentation

**Fig. 1:**
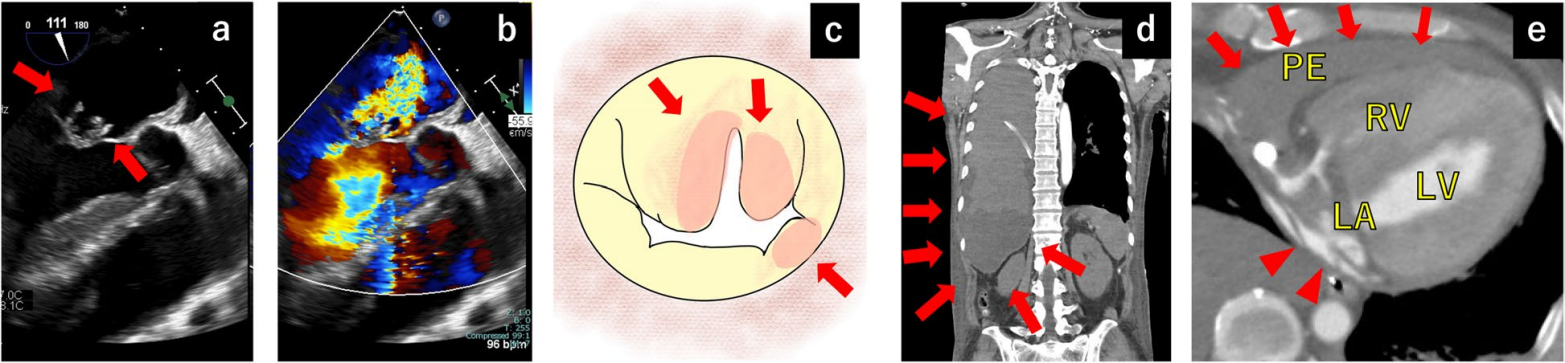
Preoperative transesophageal echocardiography, intraoperative findings, and postoperative contrast-enhanced computed tomography findings. **a**, **b** TEE shows severe mitral regurgitation with vegetation on both the anterior and posterior leaflets (A2, P3) (red arrows). **c** The vegetation was attached to the leaflets A2, A3, and P3, and there was also an abscess formation at the annulus, especially from P3 to the posterior commissure (red arrows). **d** and **e** Contrast-enhanced computed tomography reveals massive hematoma in the right thoracic cavity and pericardium (red arrows) and the extravasation from the left atrium (arrowheads). RV, right ventricle; LA, left atrium; LV, left ventricle; PE, pericardial effusion

A 60-year-old man was admitted to our hospital with bilateral lower-extremity purpura and worsening cognitive decline over the past 6 months. Chest radiography revealed an enlarged cardiac silhouette and pulmonary venous congestion. Laboratory tests revealed an elevated white blood cell count of 15,400/μL, serum creatinine level of 4.90 mg/dL, blood urea nitrogen level of 74 mg/dL, and C-reactive protein level of 12.8 mg/dL. Blood cultures on admission grew *Streptococcus mitis*. Transesophageal echocardiography (TEE) showed severe mitral regurgitation (MR) with vegetation on both mitral leaflets (A2 and P3) (Fig. 1a, b). We diagnosed IE with severe MR. Brain computed tomography (CT) revealed a subacute hemorrhagic infarction in the right parietal lobe.

**Fig. 2:**
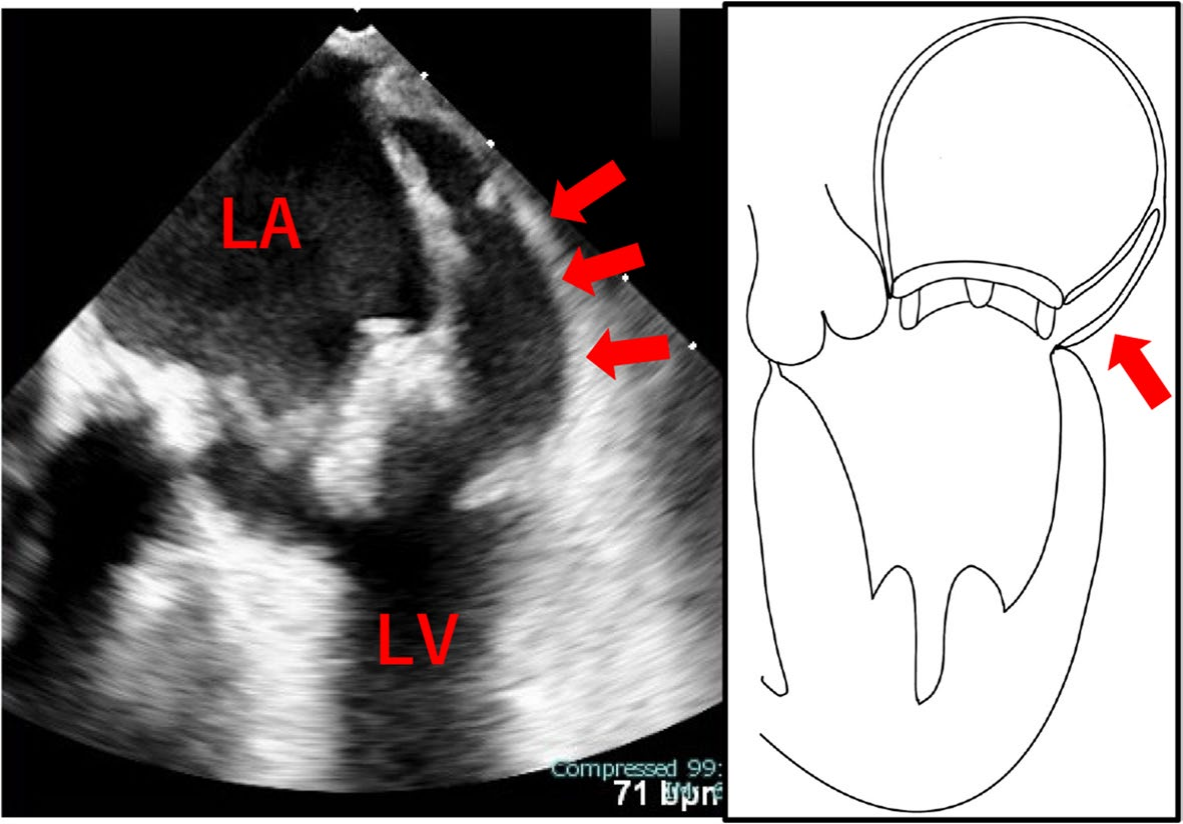
Transesophageal echocardiography (TEE) reveals the prosthetic valve partial detachment and a false lumen posterior side of left atrium, where blood flows into

We planned to perform MVR 2 weeks after the antibiotic injections because the patient had cerebral hemorrhagic complications. He underwent minimally invasive cardiac surgery-MVR with a bioprosthetic valve 29 mm (Epic; Abbott Laboratories, Green Oaks, IL, USA) using everting mattress suture technique; vegetations were detected on both leaflets (A2, A3, and P3), and the mitral annulus was fragile with abscess formation from P3 to the posterior commissure. The patient was admitted to the intensive care unit (ICU) in stable condition with minimal inotropic support. The patient recovered uneventfully, was extubated the following day, and was discharged from the ICU 2 days after the operation. On postoperative day (POD) 3, the patient experienced sudden cardiac arrest (pulseless electrical activity), and cardiopulmonary resuscitation was immediately initiated. A percutaneous venoarterial extracorporeal membrane oxygenation (VA-ECMO) cannula was inserted emergently, and the patient achieved a return of spontaneous circulation. Contrast-enhanced CT showed that the mediastinum and right thoracic cavity were filled with a massive hematoma (Fig. 1d, e). Accordingly, we performed an emergency median sternotomy and provided hemostasis for the main bleeding from the posterior wall of the left atrium. We sutured the posterior wall of the left atrium and performed compression hemostasis with bovine pericardium using a surgical sealant (AQUABRID; Hydrofit, Terumo, Tokyo, Japan) for active bleeding from the left atrium. Coagulability was disrupted; therefore, the patient was returned to the ICU with gauze packing. When we retrieved the gauze 2 days after packing, TEE showed a false lumen in the posterior aspect of the left atrium, and instability of the valve annular and left atrial dissection was diagnosed (Fig. 2). To manage the prosthetic valve detachment and concerns regarding rebleeding from the false lumen, we planned to perform redo MVR with a median sternotomy. While waiting for tissue stabilization, we decided to perform redo MVR 5 days later.

**Fig. 3:**
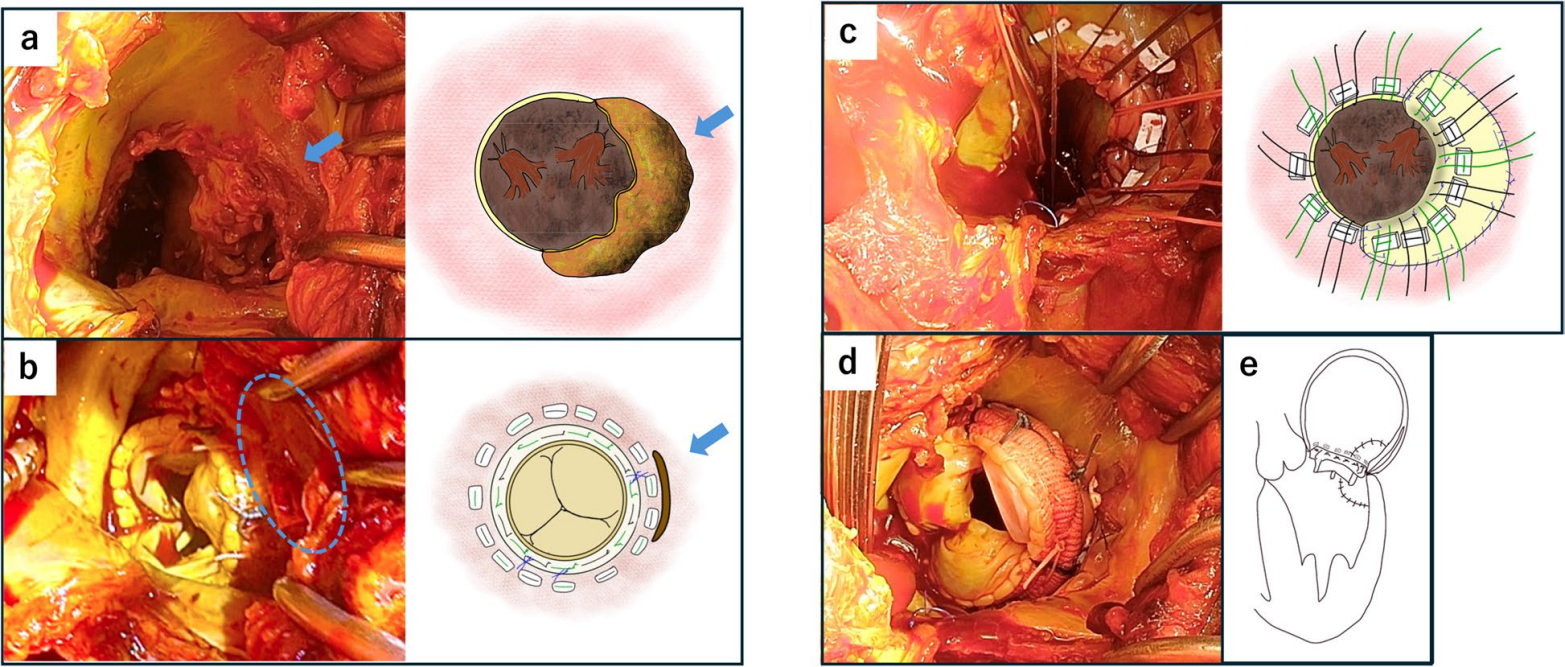
Intraoperative findings. **a** Upon removing the prosthetic valve and observing the mitral valve, a tear is observed in the mitral annulus at 3 o’clock, forming an entry. **b** The entry point was observed on the outer side of the prosthetic valve and the pledget (blue circle and blue arrow). **c**, **d** The valve annulus is fragile, and we performed annular reconstruction with a bovine pericardial patch from 2 o’clock to 6 o’clock to cover the tear and reinforce the valve annulus. The deepest region was sutured into the left ventricular myocardium with a gentle tension and a close suture. Valve sutures are placed in the bovine pericardium and intact annulus, and MVR is performed using Epic mitral valve 27 mm (Abbott Laboratories, Green Oaks, IL, USA). **e** The schema demonstrates the bioprosthetic mitral valve with the annular reconstruction using bovine pericardium

During redo MVR, a tear at the mitral annulus (3-o’clock position) was identified. Annular reconstruction was performed using a bovine pericardial patch from the 2-o’clock to 6-o’clock positions to cover the tear and reinforce the valve ring with mattress and running 5–0 polypropylene suture on the left atrial side and with running suture on the left ventricular side. The deepest region was sutured into the left ventricular myocardium; however, tension was not applied excessively, and a close suture was deemed sufficient to block blood flow to the defect. Valve sutures were placed in the bovine pericardium and intact annulus, and MVR was performed using Epic mitral valve 27 mm (Fig. 3). Bovine pericardium with BioGlue (CryoLife International Inc., Kennesaw, GA, USA) was applied to the left atrial wall to reinforce the wall of the false lumen. The patient was returned to the ICU on VA-ECMO. He was weaned off VA-ECMO on POD 15. Follow-up TEE on POD 41 showed resolution of the left dissection. The patient was discharged on POD 74.

## Discussion and conclusions

The incidence of left atrial dissection is reportedly 0.16% for all mitral valve procedures and 0.02% for single coronary artery bypass grafting (CABG) procedures [1].

The causes can be divided into nonsurgical and surgical [1], with mitral valve surgery accounting for 56% [1] of cardiac surgical cases and other surgical procedures such as CABG [3], aortic valve replacement [4], left ventricular aneurysm resection [5], pulmonary vein cannulation [6], and intracardiac tumor resection [7]. Nonsurgical complications include catheter ablation [8], percutaneous coronary intervention (PCI) [9], non-PCI myocardial infarction [10], and blunt cardiac trauma [10]. The onset and timing of diagnosis are reportedly within 1 day after MVR [11] or PCI [9].

Most (80.8%) left atrial dissections occur in the posterior leaflet of the left atrium [1]. The attachment between the posterior leaflet and the annulus is predominantly muscular, with little fibrous tissue. Moreover, calcification is more common in the posterior annulus, which may account for the higher incidence of left atrial dissection in the posterior annulus. Tissue fragility, such as that seen in IE, mitral annular calcification, and corticosteroid users, is a risk factor of left atrial dissection [1, 2]. Surgical factors include debridement of the excess valve annulus, selection of an oversized valve, and inadvertent traction, similar to the factors in the development of left ventricular rupture [12]. In this case, the initial MVR was performed under a video-assisted procedure, the view was not poor, and the anatomy was not difficult to suture. Although it is possible that excessive traction may be applied during suturing, previous reports have not mentioned that MICS increases the frequency of left atrial dissection, and we hypothesized that the cause of the disorder was a fragile valve annulus caused by IE.

There is no established treatment strategy for left atrial dissection due to its rarity and variable clinical presentations, ranging from asymptomatic to fatal. With the widespread use of TEE, asymptomatic left atrial dissection has become more common in recent years [13]. When circulation is stable, there are reports of cases wherein the false lumen has been reduced by the conservative approach of discontinuing anticoagulants [14]. In contrast, patients with hemodynamic instability require surgical repair. Hemodynamic instability is thought to be caused by low-output syndrome due to pulmonary vein compression and mitral inflow obstruction by the false left atrial lumen [11]. Although the key to surgical success is the removal of the hematoma, obliteration of the false lumen, and closure of the entry point, there is no consensus regarding the surgical procedure [3]. When closing the entry point, reinforcement of the fragile mitral annulus with BioGlue (CryoLife International Inc.) or bovine pericardium is reportedly helpful [1].

In this case, the patient had circulatory collapse due to rupture of the false lumen and massive hemorrhage, and he was rescued with compression hemostasis; however, TEE 2 days after the event revealed valve instability and residual flow into the false lumen. At that time, although the fatal bleeding was under control and prosthetic valve function was preserved, surgical intervention was deemed necessary owing to the risk of re-rupture of the false lumen and compression of the pulmonary vein.

Accordingly, we closed the entry point and performed redo MVR. This approach was used with reference to a reported case of MVR with annular reconstruction for IE with annular abscess [15]. They reported that a patch can be used to replace the mitral annulus when the annulus is vulnerable to IE or severe mitral annular calcification [15]. In this case, the prosthetic valve was removed, and the annulus was determined to be the defect that formed the entry point. The same area was required for repair. A bovine pericardial patch was used to provide adequate margins around defects. The oversized pericardial patch was partially sutured to the left ventricular myocardium under gentle tension. Valve sutures were placed in the intact annulus and bovine pericardium. We carefully sized and selected a valve smaller than the previous one. TEE immediately after the procedure showed that blood flow into the false lumen had disappeared. On the other hand, when the annulus is judged to be fragile intraoperatively, such as in cases of IE, annular reconstruction with a bovine pericardial patch may reduce the incidence of left atrial dissection.

In conclusion, we report a case of left atrial dissection following MVR. The complex lesion was successfully repaired using redo MVR with annular reconstruction.

## Data Availability

The datasets supporting the conclusions of this article are included within the article.

## Abbreviations

CABG: Coronary artery bypass grafting
CT: Computed tomography
ICU: Intensive care unit
IE: Infective endocarditis
MVR: Mitral valve replacement
PCI: Percutaneous coronary intervention
POD: Postoperative day
TEE: Transesophageal echocardiography
VA-ECMO: Venoarterial extracorporeal membrane oxygenation
